# Changes in P2Y Purinergic Receptor Expression in the Ciliary Body in a Murine Model of Glaucoma

**DOI:** 10.3389/fphar.2017.00719

**Published:** 2017-10-10

**Authors:** Begoña Fonseca, Alejandro Martínez-Águila, María J. Pérez de Lara, Maria Teresa Miras-Portugal, Rosa Gómez-Villafuertes, Jesús Pintor

**Affiliations:** ^1^Departamento de Bioquímica y Biología Molecular IV, Facultad de Óptica y Optometría, Universidad Complutense de Madrid, Madrid, Spain; ^2^Departamento de Bioquímica y Biología Molecular IV, Facultad de Veterinaria, Universidad Complutense de Madrid, Madrid, Spain

**Keywords:** Ap_4_A, ciliary body, DBA/2J, eye, glaucoma, P2Y receptors

## Abstract

Glaucoma is a neuropathology, often accompanied by an elevated intraocular pressure (IOP), which can lead to blindness. Since DBA/2J mice develop glaucoma, several studies of the physiopathology of glaucoma have been reported in this animal model. It is also known that purinergic receptors are involved in the pathology of glaucoma by controlling aqueous humor production and drainage and therefore controlling IOP. There are no studies on purinergic receptors in the DBA/2J model of glaucoma and their relation to the development of the pathology, so the aim of this study was to make an approach to the purinergic mechanisms involved in glaucoma. All the experiments were performed using DBA/2J and C57BL/6J mice and investigating P2Y_1_, P2Y_2_, and P2Y_6_ receptors. IOP measurements were made with a non-invasive rebound tonometer, and animals were instilled with diadenosine tetraphosphate (Ap_4_A) and the corresponding purinergic antagonists in order to see their effects on IOP. The expression of mRNA for P2Y_1_, P2Y_2_, and P2Y_6_ purinergic receptors was carried out by quantitative real-time PCR. Additionally, P2Y-receptor expression was performed by immunohistochemical techniques carried out on the ciliary processes. The results showed that IOP decreases when Ap_4_A was instilled and that the expressions of the analyzed purinergic receptors were stable throughout all the ages under study in the C57BL/6J mice (control mice). On the other hand, there were significant changes in the purinergic receptor expression in DBA/2J suggesting that elevated IOP in these animals could be related to an increase of P2Y_2_ expression and a decrease in P2Y_1_ receptors.

## Introduction

DBA/2J mouse strain has become a popular model for studying glaucoma, because it develops the pathology spontaneously. DBA/2J mice contain mutations in two genes, *Tyrp1* and *Gpnmb*, encoding tyrosinase-related and glycosylated transmembrane proteins, respectively. These mutations lead to pigment dispersion, iris transillumination, iris atrophy, and anterior synechia (Anderson et al., 2002). Due to the blockade of aqueous outflow, DBA/2J mice suffer from ocular hypertension by the age of 9 months, which is accompanied by the canonical symptoms of a glaucoma-related death of retinal ganglion cells (RGCs), optic nerve atrophy and cupping, as well as visual deficits (Libby et al., 2005). The pathophysiology of glaucoma remains in part unknown, although there are several studies demonstrating that elevated intraocular pressure (IOP) affects disease development, leading to a progressive optic neuropathy characterized by functional and structural impairment of ocular tissues that may result in the loss of vision (Davson, 1993). This elevated IOP mainly leads to glaucoma as a result of impeded aqueous humor outflow (Tomarev, 2001). Aqueous humor is produced by the ciliary epithelium in the posterior chamber of the eye and circulates through the pupil to the anterior chamber, where it drains through the trabecular meshwork into Schlemm’s canal and episcleral veins (Morrison and Acott, 2003). Thus, the ciliary processes provide the pressure inside the eye, which is maintained as a balance between the production and the drainage of the aqueous humor throughout the trabecular meshwork.

It is well known that purinergic P2 receptors, which are activated by extracellular nucleotides, can be involved in aqueous humor production and drainage and are therefore involved in IOP control (Guzman-Aranguez et al., 2013). P2 receptors are classified into two subfamilies: G protein-coupled P2Y receptors and ligand-gated cation channels called P2X receptors. So far, seven P2X subunits (P2X1-7) and eight P2Y receptors (P2Y_1,2,4,6,11,12,13,14_) have been cloned and characterized in humans, according to their agonist sensitivity, sequence identities, and signal transduction mechanisms (Burnstock, 2000; Abbracchio et al., 2006). P2Y receptors contain seven hydrophobic transmembrane domains connected by three extracellular loops and three intracellular loops. The extracellular amino-terminus presents sites for glycosylation and the intracellular domain contains potential sites for phosphorylation, which may participate in receptor desensitization and internalization. P2Y receptors can be characterized according to their responses to nucleotide agonists and subtype-preferring antagonists. Adenine nucleotides, such as ADP or its synthetic analogous 2-methylthio-ADP (2-MeSADP), can activate selectively P2Y_1_ receptors (Waldo and Harden, 2004), whereas uracil nucleotides activate P2Y_6_ receptors (Nicholas et al., 1996). Moreover, both adenine and uracil nucleotides (ATP and UTP) activate equipotently P2Y_2_ receptors (Lazarowski et al., 1995).

P2Y_1_ and P2Y_2_ receptors are expressed in rabbit ciliary body epithelial cells, which may be responsible for the action of 2-MeSATP, ATPγS, as well as other P2Y agonists (Farahbakhsh and Cilluffo, 2002). The expression of P2Y_1_, P2Y_2_, and P2Y_4_ receptors in bovine trabecular meshwork has also been reported (Soto et al., 2005) as well as the presence of P2Y_6_ and P2Y_11_ receptors in rat ocular structures (Pintor et al., 2004).

The dinucleotide diadenosine tetraphosphate (Ap_4_A) (Guzman-Aranguez et al., 2011) shows a hypotensive effect when instilled topically in the eye (Fonseca et al., 2016), which is related to an increase in the outflow of the aqueous humor through activation of several purinergic receptors, mainly the P2Y_1_ (Schachter et al., 1996; Soto et al., 2005).

Most of the experiments performed to analyze the modulation of aqueous humor production and drainage by purinergic receptors previously described in the scientific literature, have been carried out on normotensive mice models or on induced models of glaucoma. Here we characterize the presence of P2Y_1_, P2Y_2_, and P2Y_6_ receptors in the ciliary processes of the eye of control versus glaucomatous mice and analyze the expression levels of these receptors during aging and the development of the pathology in these animals.

## Materials and Methods

### Animals

Experiments were performed on adult female C57BL/6J (*n* = 30) (control animals) and DBA/2J (*n* = 30) (glaucomatous animals) mice obtained from the European distributor of Jackson Laboratories Mice (Charles River Laboratories). All animal maintenance and experimental procedures followed Spanish and European guidelines for animal care in the laboratory and animal research (Guide for the Care and Use of Laboratory Animals) and the ARVO Statement for the Use of Animals in Ophthalmic and Vision Research. Mice were housed (1–4 mice per cage) in temperature and light-controlled rooms maintained according to a 12-h/12-h light/dark cycle; all animals were fed *ad libitum*. DBA/2J and C57BL/6J mice were studied at 3, 6, 9, and 12 months of age.

All this study has been approved by the Animal Experimental Committee of the Universidad Complutense de Madrid and Comunidad de Madrid, reference 45/057949.9/16.

### Intraocular Pressure (IOP) Measurements

Intraocular pressure (IOP) was measured using a non-invasive rebound tonometer (Tono-lab^®^; Tiolat, OY, Helsinki, Finland). The tonometer was fixed so that the probe tip was aligned with the optical axis of the eye, at a distance of 1–4 mm. In order to avoid the effect of the circadian rhythm, the IOP was always tested at the same time of day. Six consecutive measurements were taken for each reading, and three readings were obtained on each eye (Fonseca et al., 2016).

In order to study the effect of Ap_4_A, two IOP measurements were taken before Ap_4_A was instilled. A single application of 2 μl drops with a micropipette at 100 μM was instilled once every hour for 6 h. To study the effect of the antagonists of purinergic receptors, these blockers were instilled in 2 μl drops with a micropipette at 100 μM 30 min before Ap_4_A at a concentration of 100 μM, measuring IOP in the same way as previously described (Fonseca et al., 2016). The antagonists assayed in the current study were pyridoxalphosphate-6-azophenyl-2’,4’-disulfonic acid (PPADS), suramin and reactive blue 2 (RB-2, a non-selective P2 antagonists), 2’-deoxy-N6-methyladenosine 3’,5’-bisphosphate tetrasodium salt (MRS2179, a P2Y_1_ antagonist), and N,N′′-1,4-butanediylbis(N′-(3-isothiocyanatophenyl))thiourea (MRS2578, a P2Y_6_ antagonist). All the doses (concentrations), volumes, and times of measurement have been controlled according to Fonseca et al. (2016).

### RNA Isolation and RT-PCR

Total RNA from isolated ciliary bodies and iris of DBA/2J (*n* = 24) and C57BL/6 (*n* = 24) mice was extracted using Speedtools total RNA extraction kit (Biotools, Madrid, Spain), following the manufacturer’s instructions. After digestion with TURBO DNase (Ambion, Austin, TX, United States), total RNA was quantified and reverse transcribed using M-MLV reverse transcriptase, 6 μg of random primers and 350 μM dNTPs (Invitrogen, San Francisco, CA, United States). Due to the small size of the tissues and in order to improve sensitivity of PCR analysis, pre-amplification reactions were carried out using DNA AmpliTools Master Mix (Biotools), 5 μL of the RT product and specific commercial oligonucleotide primers for mouse P2Y_1_, P2Y_2_, and P2Y_6_ receptors (Applied Biosystems). Moreover, non-template control was amplified to check for contamination during the procedure. Pre-amplification reactions were performed on a 2720 Thermal Cycler (Applied Biosystems) with the following program: initial denaturation step at 94°C for 5 min followed by 14 cycles of amplification (94°C for 30 s, 60°C for 30 s, and 72°C for 30 s). Then, 5 μL of the pre-amplified product diluted 1/5 in water was used for the subsequent PCR assay. PCR reactions were identical to pre-amplification ones, with the exception of the number of cycles, which were 40 in the last case. Amplified PCR products were electrophoresed on a 2% agarose gel and visualized by SYBR^®^ Safe DNA gel stain (Invitrogen). DNA ladders used were GeneRuler 1 Kb and 100 bp (Thermo Scientific).

### Quantitative Real-Time PCR

Following pre-amplification, 5 μL of the each product diluted 1/5 in water were used for the subsequent quantitative real-time PCR (Q-PCR) assay. Q-PCR reactions were carried out using LuminoCt^®^ Q-PCR Readymix (Sigma–Aldrich, St. Louis, MO, United States), 5 μL of the RT product, and specific commercial oligonucleotide primers and TaqMan MGB probes for mouse P2Y_1_, P2Y_2_, and P2Y_6_ receptors, as well as for GAPDH (all from Applied Biosystems). Fast thermal cycling was performed using a StepOnePlus^®^ Real-Time System (Applied Biosystems, Foster City, CA, United States) as follows: denaturation, one cycle of 95°C for 20 s, followed by 40 cycles each of 95°C for 1 s and 60°C for 20 s. The results were normalized as indicated by the parallel amplification of the GAPDH housekeeping gene. Thus, Q-PCR graphs indicate the ratio between P2Y and GAPDH transcripts multiplied by the 10e4 factor in order to represent *Y*-axis values higher than 1.

### Immunohistochemistry

C57BL/6J and DBA/2J mice at 3 and 12 months of age (*n* = 6 animals for each group) were euthanized with an intraperitoneal injection overdose of pentobarvital (Dolethal; Vetoquinol^®^; Especialidades Veterinarias, S.A., Alcobendas, Madrid, Spain) and perfused pericardially with phosphate buffer saline (PBS) followed by a solution of 4% paraformaldehyde in PBS 0.1 M, pH 7.4 at 4°C. The eyes were enucleated and dissected with curved forceps and sterile scissors. The anterior pole was immersed in paraformaldehyde (PFA) fixative solution for 1 h at 4°C and was washed in PBS, and rinsed in 11% sucrose solution for 1 h and 33% sucrose solution overnight at 4°C as cryoprotection procedure. Finally, the structures were embedded in tissue medium freezing medium (Tissue-Tekaaa OCT) using liquid N_2_ and vertical sections (10 μm thick) were cut on a cryostat (Microm, Walldorf, Germany) and collected on poly-L lysine-coated slides and stored at -20°C until use.

Frozen sections were rinsed in PBS 1× and permeabilized with PBS-containing 0.25% Triton X-100 (TX-100) for 30 min. To avoid non-specific staining, sections were incubated with the blocking solution containing 10% normal donkey serum (NDS; Jackson ImmunoResearch, West Grove, PA, United States) and 0.1% TX-100 in PBS for 1 h at room temperature. Then, the following primary antibodies diluted in PBS containing 0.1% TX-100 were incubated at 4°C overnight: goat anti-P2Y_1_ (Santa Cruz Biotechnology, Inc., Santa Cruz, CA, United States; sc-15204, 1:100), rabbit anti-P2Y_2_ (Alomone Labs Israel, APR-010, 1:100) goat anti-P2Y_6_ (Santa Cruz Biotechnology, Inc., Santa Cruz, CA, United States; sc-15215, 1:75) diluted in PBS-0.1% TX-100 were incubated at 4°C overnight. Finally, tissue sections were washed in PBS containing 0.1% TX-100 and incubated. The secondary antibody donkey anti-rabbit Alexa Fluor 488 IgG (H+L) (Jackson ImmunoResearch, West Grove, PA, United States) was diluted 1:200 in PBS containing 0.1% TX-0.1% for 1 h in darkness at room temperature. Nuclei were stained with propidium iodide (red, Sigma–Aldrich, St. Louis, MO, United States) diluted 1:500 in PBS for 10 min. Finally, sections were rinsed and mounted in Vectashield (Vector Laboratories, Palex Medical, Barcelona, Spain) and coverslipped. Negative controls were carried out by following the same procedures but, in each case, the primary antibody was substituted by the same volume of PBS/TX-100 solution. For the analysis, the images were acquired using a laser-scanning microscope (Zeiss LSM 5, Jena, Germany) at 40× magnification and exported as tiff files for further analysis.

### Statistical Analysis

All data are presented as the mean ± SEM. Statistical differences were calculated using one-way ANOVA test with Dunnett’s post-test plotting and fitting were carried out by GraphPad Prism 6 computer program (GraphPad Software).

## Results

### Activation of P2Y Receptors Decreases IOP in C57BL/6J and DBA/2J Mice Strains

**FIGURE 1:**
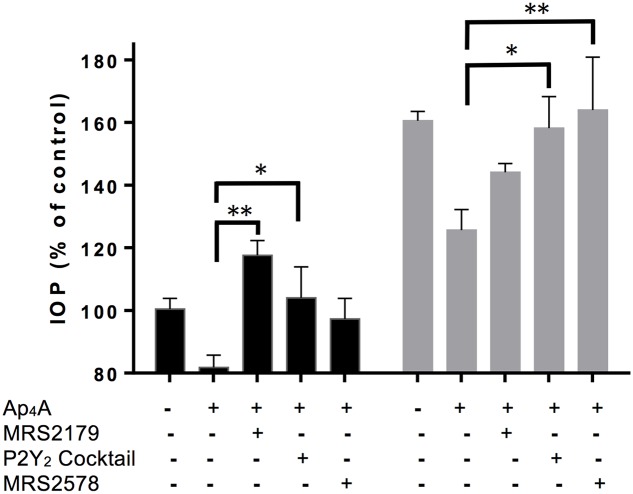
Effect of P2 receptor antagonists on IOP reduction mediated by Ap_4_A in C57BL/6J versus DBA/2J mice. Two IOP measurements were taken before a single dose of either vehicle or 100 μM Ap_4_A was instilled, and 3 h later, IOP was measured again. When indicated, purinergic antagonists, all tested at 100 μM, were instilled 30 min before Ap_4_A. MRS2179 and MRS2578 are antagonists of P2Y_1_ and P2Y_6_ receptors, respectively. “P2Y_2_ cocktail” contains PPADS, suramin, and RB-2. Data are the mean ± SEM of 4 mice (^∗^*p* < 0.05, ^∗∗^*p* < 0.01; one-way ANOVA with Dunnett’s multiple comparisons test).

**FIGURE 2:**
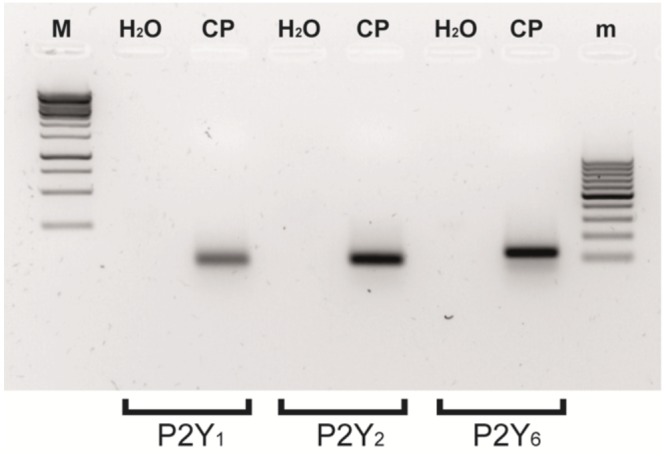
Several metabotropic P2Y receptors are simultaneously expressed in the eye ciliary processes of C57BL/6J mice. RT-PCR expression of P2Y_1_, P2Y_2_, and P2Y_6_ receptors. Molecular weights of the bands were around 100 bp and were amplified from adult C57BL/6J mouse ciliary processes (CP) mRNA extracts. No amplification products were observed in parallel assays carried out without template (H_2_O). M: 1 Kb DNA ladder (10000-250 pb); m: 100 bp DNA ladder (1000–1100 bp).

Ap_4_A has been reported as an agonist of P2Y_1_, P2Y_2_, and P2Y_6_ receptors (Patel et al., 2001; Pintor et al., 2002). In a previous study, we have demonstrated that the instillation of Ap_4_A was able to significantly decrease IOP in both control C57BL/6J and glaucomatous DBA/2J aged mice, with a maximal effect after 3 h of treatment (Fonseca et al., 2016). In the current study, we tested the effect of different P2Y receptor antagonists on the IOP reduction mediated by Ap_4_A in 12-month-old C57BL/6J versus DBA/2J mice (**Figure 1**). It was noticed that the Ap_4_A effect on control animals was completely blocked by either MRS2179, a P2Y_1_ antagonist (^∗∗^*p* < 0.005), or a cocktail of suramin, PPADS and RB-2, which altogether inhibits P2Y_2_ receptor (^∗^*p* < 0.05), whereas MRS2578, a P2Y_6_ antagonist, did not show a significant effect on the Ap_4_A hypotensive effect. On the contrary, in glaucomatous animals, the P2Y_1_ antagonist did not modify the Ap_4_A effect, whereas either the “P2Y_2_ cocktail” or the P2Y_6_ antagonist MRS2578 was able to prevent the IOP fall induced by Ap_4_A. We have also performed RT-PCR experiments demonstrating that P2Y_1_, P2Y_2_, and P2Y_6_ transcripts were expressed in the ciliary processes of C57BL/6J mice (**Figure 2**). These results suggested that the expression levels of P2Y receptors could differ between control and glaucomatous animals. In order to check this possibility, we analyzed, by Q-PCR and immunohistochemistry, the expression levels of P2Y_1_, P2Y_2_, and P2Y_6_ receptors in the ciliary processes of both C57BL/6J and DBA/2J mice at different ages.

### Expression of P2Y_1_ Receptors in Ciliary Processes of C57BL/6J and DBA/2J Mice

**FIGURE 3:**
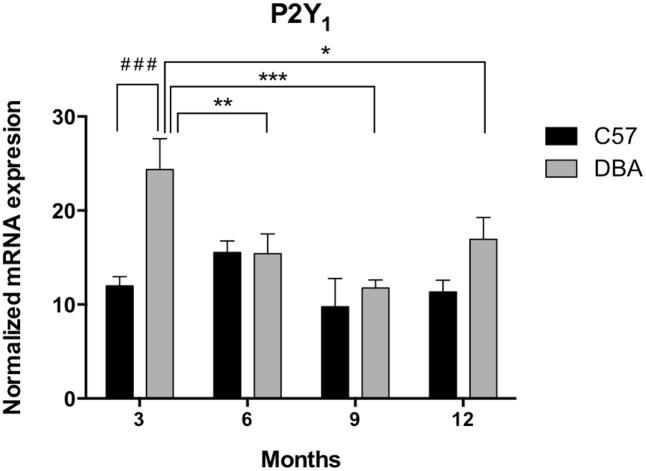
Temporal pattern of P2Y_1_ transcript expression in ciliary processes of C57BL/6J versus DBA/2J mice. Total RNA from ciliary processes of either control (C57BL/6J) or glaucomatous (DBA/2J) animals of 3, 6, 9, or 12 months of age was extracted and P2Y_1_ mRNA was quantified by Q-PCR as described in the Section “Materials and Methods.” Values were normalized to the content of GAPDH transcript. Results are the mean ± SEM of 24 animals of each strain (^∗^*p* < 0.05, ^∗∗^*p* < 0.01, ^∗∗∗^*p* < 0.001 versus same mice strain; ^###^*p* < 0.001 versus different mice strain; two-way ANOVA with Sidak’s post-test).

**FIGURE 4:**
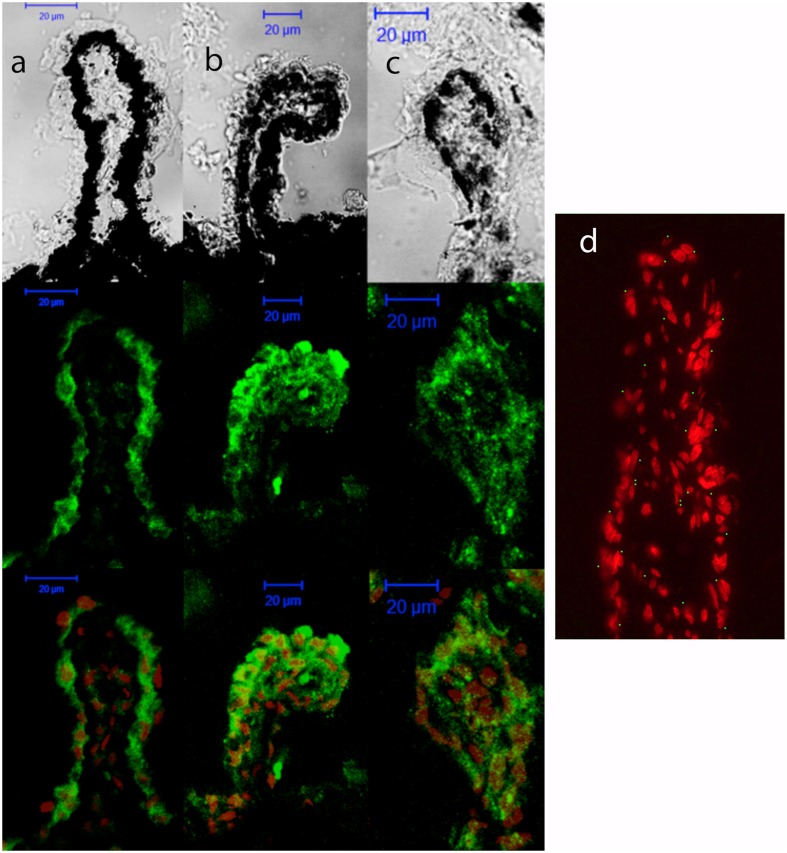
Cellular distribution of P2Y_1_ receptor in ciliary processes of C57BL/6J versus DBA/2J mice. Immunofluorescence images of ciliary processes from 3-month-old C57BL/6J **(a)**, 3-month-old DBA/2J **(b)**, and 12-month-old DBA/2J **(c)** mice labeled with antibodies against P2Y_1_ receptor (green). Nuclei were counterstained with propidium iodide (red). **(d)** Negative control was carried out by substituting the P2Y_1_ primary antibody by the same volume of PBS/TX-100 solution. Phase-contrast and confocal images show that P2Y_1_ immunostaining is mainly located in non-pigmented epithelium of ciliary processes, being enhanced in DBA/2J versus C57BL/6J at the age of 3 months. Scale bar: 20 μm.

First, the expression levels of P2Y_1_ transcript were compared between the control and glaucomatous mice of 3, 6, 9, and 12 months of age by Q-PCR. In C57BL/6J mice, P2Y_1_ mRNA expression remained invariable during the adult life of the animals (**Figure 3**, black bars). Interestingly, P2Y_1_ expression in DBA/2J mice was twofold higher than in C57BL/6J animals at 3-month-old (*p* = 0.005), but decreased to control values in older mice (**Figure 3**, gray bars). Immunohistochemical studies revealed that the P2Y_1_ receptor was widely distributed over all in the non-pigmented epithelium of the ciliary process of the eye in both C57BL/6 and DBA/2J mice. However, as observed in transcript levels, P2Y_1_ receptor expression was enhanced in 3-month-old DBA/2J animals compared to 12-month-old ones and also compared to 3-month-old C57BL/6 mice (**Figure 4**).

### Expression of P2Y_2_ Receptors in Ciliary Processes of C57BL/6J and DBA/2J Mice

**FIGURE 5:**
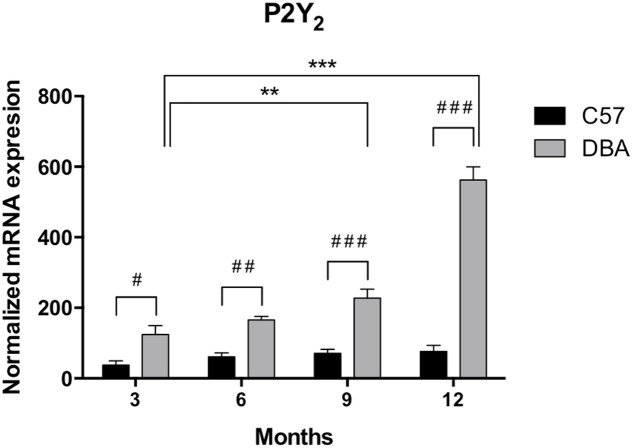
Temporal pattern of P2Y_2_ mRNA levels in ciliary processes of C57BL/6J versus DBA/2J mice. Total RNA from ciliary processes of either control (C57BL/6J) or glaucomatous (DBA/2J) animals of 3, 6, 9, or 12 months of age was extracted and P2Y_2_ mRNA was quantified by Q-PCR as described in the Section “Materials and Methods.” GAPDH was used as a control for differences in cDNA input. Results are the mean ± SEM of 24 animals of each strain (^∗∗^*p* < 0.01, ^∗∗∗^*p* < 0.001 versus same mice strain; ^#^*p* < 0.05, ^##^*p* < 0.01 ^###^*p* < 0.001 versus different mice strain; two-way ANOVA with Sidak’s post-test).

**FIGURE 6:**
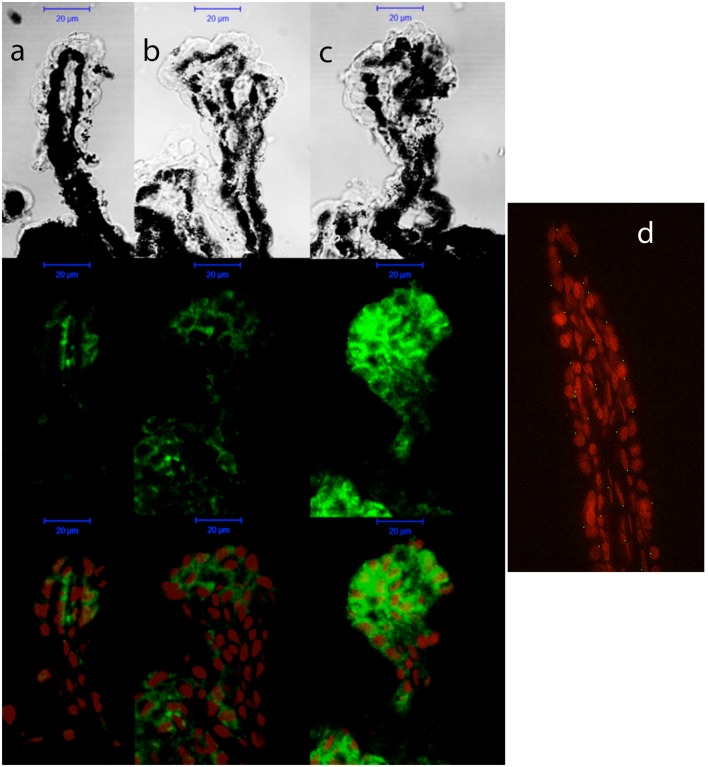
Cellular distribution of P2Y_2_ receptor in ciliary processes of C57BL/6J versus DBA/2J mice. Immunofluorescence images of ciliary processes from 3-month-old C57BL/6J **(a)**, 3-month-old DBA/2J **(b)**, and 12-month-old DBA/2J **(c)** mice labeled with antibodies against P2Y_2_ receptor (green). Nuclei were counterstained with propidium iodide (red). **(d)** Negative control carried out by substituting the P2Y_2_ primary antibody by the same volume of PBS/TX-100 solution. Phase-contrast and confocal images show that the P2Y_2_ receptor is mainly located in the non-pigmented epithelium of ciliary processes, and its expression is strongly increased in old DBA/2J mice compared to young animals. Scale bar: 20 μm.

As shown for P2Y_1_ transcript, P2Y_2_ mRNA expression remained invariable during the adult life of C57BL/6J mice (**Figure 5**, black bars). However, a pathology-dependent rise in P2Y_2_ transcript levels was found in glaucomatous mice, reaching a sixfold increase in 12-month-old DBA/2J mice compared to control ones (*p* = 0.005), although a significant increase was already observed in 6-month-old mice (**Figure 5**, gray bars). P2Y_2_ protein was also found in the non-pigmented epithelium of the ciliary processes of both C57BL/6J and DBA/2J animals, although, as expected, a huge increment in the fluorescent signal coupled to P2Y_2_ receptor was found in 12-month-old DBA/2J mice compared to young animals of both strains (**Figure 6**).

### Expression of P2Y_6_ Receptors in Ciliary Processes of C57BL/6J and DBA/2J Mice

**FIGURE 7:**
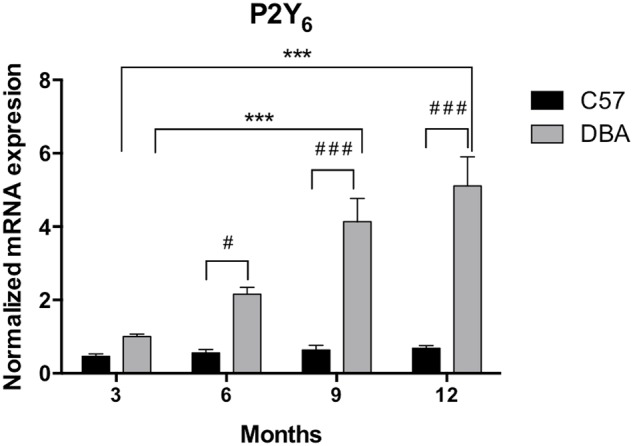
Temporal pattern of P2Y_6_ transcript expression in ciliary processes of C57BL/6J versus DBA/2J mice. Total RNA from ciliary processes of either control (C57BL/6J) or glaucomatous (DBA/2J) animals of 3, 6, 9, or 12 months of age was extracted and P2Y_6_ mRNA was quantified by Q-PCR as described in the Section “Materials and Methods.” Values were normalized to the content of GAPDH transcript. Results are the mean ± SEM of 24 animals of each strain (^∗∗∗^*p* < 0.001 versus same mice strain; ^#^*p* < 0.05, ^###^*p* < 0.001 versus different mice strain; two-way ANOVA with Sidak’s post-test).

**FIGURE 8:**
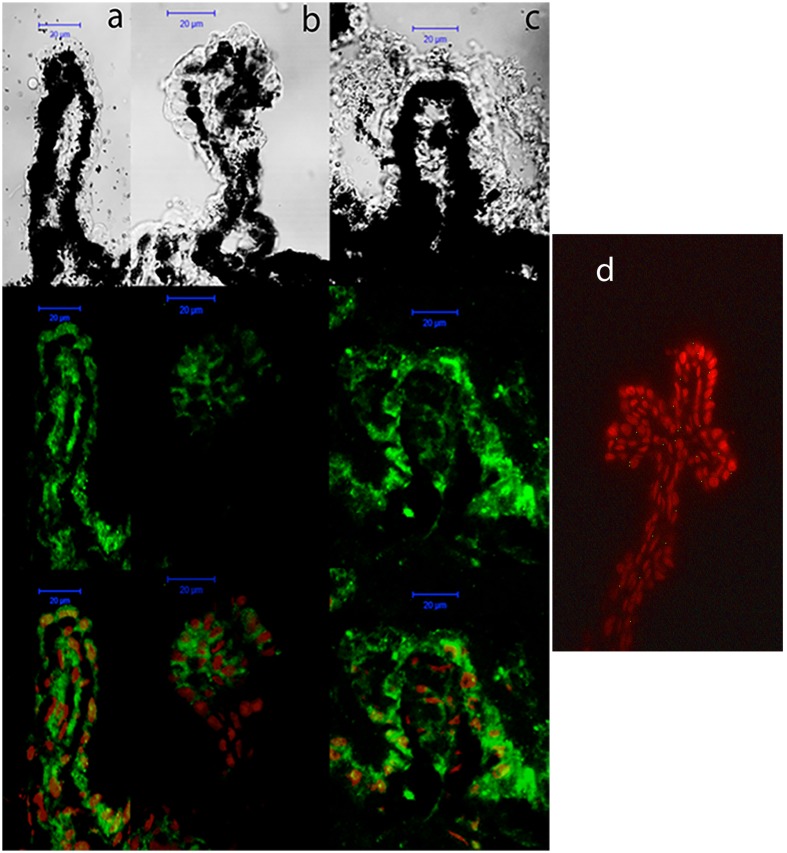
Cellular distribution of P2Y_6_ receptor in ciliary processes of C57BL/6J versus DBA/2J mice. Immunofluorescence images of ciliary processes from 3-month-old C57BL/6J **(a)**, 3-month-old DBA/2J **(b)**, and 12-month-old DBA/2J **(c)** mice labeled with antibodies against P2Y_6_ receptor (green). Nuclei were counterstained with propidium iodide (red). **(d)** Negative control was carried out by substituting the P2Y_6_ primary antibody by the same volume of PBS/TX-100 solution. Phase-contrast and confocal images show that the P2Y_6_ receptor is mainly located in the non-pigmented epithelium of ciliary processes, and its expression is increased in 12-month-old DBA/2J mice compared to 3-month-old animals. Scale bar: 20 μm.

The expression pattern of P2Y_6_ receptors was very similar to that observed for P2Y_2_ receptors. As shown in **Figure 7**, P2Y_6_ transcript levels remained constant in C57BL/6J mice (black bars), but an age-dependent increase in P2Y_6_ mRNA was observed in *DBA/2J* mice (gray bars). This increment was significant in 6-month-old animals and was progressively rising until reaching a sixfold increase in 12-month-old DBA/2J mice compared to control ones (*p* = 0.005). Immunohistochemical studies confirmed that the P2Y_6_ receptor was also located in the non-pigmented epithelium of the ciliary body of the eye of both mice strains. Moreover, as observed in transcript levels, P2Y_6_ receptor expression was significantly higher in 12-month-old DBA/2J animals compared to young ones and also compared to young C57BL/6 mice (**Figure 8**).

## Discussion

The existence of nucleotides in the aqueous humor (Mitchell et al., 1998; Pintor et al., 2003), as well as the participation of P2 purinergic receptors in the regulation of IOP, has already been described in the scientific literature (Crooke et al., 2008). Mono and dinucleotides, both in the μM range, are able to activate P2 receptors, which in turn modify both the synthesis and drainage of the aqueous humor. In normotensive animal models, P2Y_1_ and P2Y_2_ receptors are located in the trabecular meshwork and the ciliary body, respectively (Soto et al., 2005; Martin-Gil et al., 2012). When the activation of P2Y_1_ receptors is carried out by Ap_4_A in the trabecular meshwork, the observed effect is a reduction in IOP (Soto et al., 2005), whereas the activation of P2Y_2_ receptors in the ciliary body produces a hypertensive effect (Martin-Gil et al., 2012). P2Y_2_ receptors exert this effect by increasing the presence of Aquaporin-1 in the ciliary body epithelial cell membranes (Martin-Gil and Pintor, 2010; Pintor et al., 2011). Finally, the activation of P2Y_6_ receptor by molecules such as uridine diphosphate (UDP), reduces IOP in the New Zealand normotensive model (Markovskaya et al., 2008).

Ap_4_A is a molecule present in the aqueous humor of animal models such as rabbits or mice (Pintor et al., 2003), but is also present in normal and glaucomatous human patients (Castany et al., 2011). Therefore, this dinucleotide seems to be involved in the pathophysiology of glaucoma, mainly by acting on P2 purinergic receptors. In this sense, the release of Ap_4_A is produced by the activation of a pressure sensor, a TRPV4 channel, which is activated by the abnormal IOP often associated to glaucoma (Pintor et al., 2011). The TRPV4-induced Ap_4_A release will stimulate mainly the P2Y_2_ receptor present in the ciliary body, which mobilizes Aquaporin-1 from intracellular reservoirs to the plasma membrane of the ciliary epithelium (Martin-Gil and Pintor, 2010). This increase in the aquaporins facilitates the production of aqueous humor contributing to the elevation of IOP and therefore priming the described process.

In this current study, using a glaucoma animal model, we have analyzed the expression levels of three P2Y metabotropic receptors (P2Y_1_, P2Y_2_, and P2Y_6_), which are known to be activated by Ap_4_A (Guzman-Aranguez et al., 2007), in the ciliary body and iris of control (C57BL/6) and glaucomatous (DBA/2J) mice. Our results demonstrate that control animals express constant levels of P2Y_1_, P2Y_2_, and P2Y_6_ receptors throughout their lives, in clear contrast to the glaucomatous strain, where more remarkable changes are observed. In this sense, P2Y_1_ receptor expression is significantly reduced at both mRNA and protein levels in aged DBA/2J mice. On the contrary, however, P2Y_2_ and P2Y_6_ receptor expressions are enhanced in aged DBA/2J mice, correlating in time with the pathology development. Similar differences between control and glaucomatous mice have also been found when analyzing the retinal electrophysiology in both strains, observing a gradual ganglion cell death in DBA/2J model (Perez de Lara et al., 2014). Moreover, the retinal ATP released increases in the glaucomatous mouse, but not in the control one when the pathology is fully established (12 months of age). In addition, the expression of the vesicular nucleotide transporter (VNUT) is significantly increased during the development of glaucoma in DBA/2J mice, reaching maximal levels at 12 months of age (Perez de Lara et al., 2015). Altogether, this evidence suggests that the alteration in the purinergic system observed in the DBA/2J is related to the pathology development and not to mice aging.

We have also established that micromolar concentrations of Ap_4_A significantly reduce IOP in DBA/2J mice, once the pathology is fully developed. Moreover, a chronic treatment with the dinucleotide for 3 months is able to ameliorate the elevation in IOP in the glaucomatous mice in a very significant manner (Fonseca et al., 2016). These results indicate that the predominant physiological effect of Ap_4_A is the reduction of IOP, although the expression of both P2Y_2_ and P2Y_6_ in DBA/2J mice is increased and both receptors mediate opposite effects on IOP (Fonseca et al., 2016).

Previous studies reported that P2Y_2_ receptor activation in the ciliary body exerted a hypertensive effect on IOP, which could be inhibited by antagonism or silencing of that receptor (Martin-Gil et al., 2012). The hypertensive role of the P2Y_2_ receptor in the ciliary body correlated well with the abnormally elevated concentration of Ap_4_A found in glaucoma patients, as previously commented (Castany et al., 2011). However, the topical application of this dinucleotide reduces IOP, so its use in the treatment of glaucoma cannot be discarded (Fonseca et al., 2016).

In our experimental model, the reduction in the expression of the P2Y_1_ receptor in glaucomatous mice could produce an elevation in IOP, since the treatment with the specific antagonist MRS2179 prevented the hypotensive effect of Ap_4_A (Fonseca et al., 2016). An increase P2Y_2_ expression could have similar consequences, since its activation exerted a hypertensive effect (Fonseca et al., 2016). Thus, the pathology-related increased in P2Y_2_ receptor expression and its activation by endogenous Ap_4_A could justify high IOP values measured in the glaucomatous mice. Interestingly, exogenous applications of high doses of Ap_4_A have hypotensive effects, probably by activating the P2Y_6_ receptor, which is elevated in the glaucomatous mice. Noteworthy is that the high Ap_4_A concentration (about 300 nM) found in the aqueous humor of glaucoma patients is unable to reduce IOP, probably because this concentration is enough to stimulate the hypertensive P2Y_2_ receptor, but insufficient to activate the hypotensive P2Y_6_, which requires micromolar concentrations (Guzman-Aranguez et al., 2007). Further studies will be necessary to clarify this refined equilibrium.

The pathogenesis of glaucoma is not fully understood, but most of the studies indicate that the level of IOP is linked to RGC’s death (Hollands et al., 2013). The rise in IOP causes mechanical stress and strain affecting eye posterior structures such as the lamina cribrosa and adjacent tissues, thus causing the deformation by compression of the lamina cribrosa. Consequently, the optic nerve axonal damage reduces the retrograde axonal transport (Burgoyne et al., 2005). This process produces the RGC’s death and the subsequent blindness. Slowing disease progression, by reducing IOP, can be suggested according to the changes in the expression of P2Y receptors sensitive to the dinucleotide Ap_4_A presented in this manuscript. A previous study has already proved the hypotensive effect of this compound on the DBA/2J glaucoma model (Fonseca et al., 2016). Therefore, the application of Ap_4_A may help to reduce IOP in humans if the changes in the P2Y receptors are similar to the ones described in the glaucomatous mice.

In summary, we have demonstrated that the expression of some P2Y receptors, P2Y_1_, P2Y_2_, and P2Y_6_, changes during the development of the glaucomatous pathology in a mouse model. The prevalence of the P2Y_2_ receptor between 9 and 12 months of age, together with the rise in Ap_4_A concentration, may be a contributing factor that helps explain why the pressure is abnormally elevated when the pathology is fully established.

## Author Contributions

BF: Contributed in PCR, immunohistochemistry. AM-Á: Contributed in PCR, immunohistochemistry. MPdL: Contributed in IOP measurements. MTM-P: Contributed in paper organization and writing. RG-V: Contributed in PCR, statistical analysis, and writing. JP: Contributed in paper design, organization, and writing.

## Conflict of Interest Statement

The authors declare that the research was conducted in the absence of any commercial or financial relationships that could be construed as a potential conflict of interest.

## References

[B1] Abbracchio M. P. Burnstock G. Boeynaems J. M. Barnard E. A. Boyer J. L. Kennedy C. (2006). International Union of Pharmacology LVIII: update on the P2Y G protein-coupled nucleotide receptors: from molecular mechanisms and pathophysiology to therapy. *Pharmacol. Rev.* 58 281–341. 10.1124/pr.58.3.3 16968944 PMC3471216

[B2] Anderson M. G. Smith R. S. Hawes N. L. Zabaleta A. Chang B. Wiggs J. L. (2002). Mutations in genes encoding melanosomal proteins cause pigmentary glaucoma in DBA/2J mice. *Nat. Genet.* 30 81–85. 10.1038/ng794 11743578

[B3] Burgoyne C. F. Downs J. C. Bellezza A. J. Suh J. K. Hart R. T. (2005). The optic nerve head as a biomechanical structure: a new paradigm for understanding the role of IOP-related stress and strain in the pathophysiology of glaucomatous optic nerve head damage. *Prog. Retin. Eye Res.* 24 39–73. 10.1016/j.preteyeres.2004.06.001 15555526

[B4] Burnstock G. (2000). P2X receptors in sensory neurones. *Br. J. Anaesth.* 84 476–488. 10.1093/oxfordjournals.bja.a013473 10823099

[B5] Castany M. Jordi I. Catala J. Gual A. Morales M. Gasull X. (2011). Glaucoma patients present increased levels of diadenosine tetraphosphate, Ap(4)A, in the aqueous humour. *Exp. Eye Res.* 92 221–226. 10.1016/j.exer.2010.12.004 21147104

[B6] Crooke A. Guzman-Aranguez A. Peral A. Abdurrahman M. K. Pintor J. (2008). Nucleotides in ocular secretions: their role in ocular physiology. *Pharmacol. Ther.* 119 55–73. 10.1016/j.pharmthera.2008.04.002 18562011

[B7] Davson H. (ed.) (1993). “The aqueous humour and the intraocular pressure,” in *Physiology of the Eye* (New York: Pergamon Press), 34–95.

[B8] Farahbakhsh N. A. Cilluffo M. C. (2002). P2 purinergic receptor-coupled signaling in the rabbit ciliary body epithelium. *Invest. Ophthalmol. Vis. Sci.* 43 2317–2325. 12091433

[B9] Fonseca B. Martinez-Aguila A. De Lara M. J. Pintor J. (2016). Diadenosine tetraphosphate as a potential therapeutic nucleotide to treat glaucoma. *Purinergic Signal.* 13 171–177. 10.1007/s11302-016-9547-y 27848070 PMC5432477

[B10] Guzman-Aranguez A. Crooke A. Peral A. Hoyle C. H. Pintor J. (2007). Dinucleoside polyphosphates in the eye: from physiology to therapeutics. *Prog. Retin. Eye Res.* 26 674–687. 10.1016/j.preteyeres.2007.09.001 17931952

[B11] Guzman-Aranguez A. Loma P. Pintor J. (2011). Focus on molecules: diadenosine tetraphosphate. *Exp. Eye Res.* 92 96–97. 10.1016/j.exer.2010.12.007 21163257

[B12] Guzman-Aranguez A. Santano C. Martin-Gil A. Fonseca B. Pintor J. (2013). Nucleotides in the eye: focus on functional aspects and therapeutic perspectives. *J. Pharmacol. Exp. Ther.* 345 331–341. 10.1124/jpet.112.202473 23504005

[B13] Hollands H. Johnson D. Hollands S. Simel D. L. Jinapriya D. Sharma S. (2013). Do findings on routine examination identify patients at risk for primary open-angle glaucoma? The rational clinical examination systematic review. *JAMA* 309 2035–2042. 10.1001/jama.2013.5099 23677315

[B14] Lazarowski E. R. Watt W. C. Stutts M. J. Boucher R. C. Harden T. K. (1995). Pharmacological selectivity of the cloned human P2U-purinoceptor: potent activation by diadenosine tetraphosphate. *Br. J. Pharmacol.* 116 1619–1627. 10.1111/j.1476-5381.1995.tb16382.x 8564228 PMC1908898

[B15] Libby R. T. Anderson M. G. Pang I. H. Robinson Z. H. Savinova O. V. Cosma I. M. (2005). Inherited glaucoma in DBA/2J mice: pertinent disease features for studying the neurodegeneration. *Vis. Neurosci.* 22 637–648. 10.1017/S0952523805225130 16332275

[B16] Markovskaya A. Crooke A. Guzman-Aranguez A. I. Peral A. Ziganshin A. U. Pintor J. (2008). Hypotensive effect of UDP on intraocular pressure in rabbits. *Eur. J. Pharmacol.* 579 93–97. 10.1016/j.ejphar.2007.10.040 18031728

[B17] Martin-Gil A. De Lara M. J. Crooke A. Santano C. Peral A. Pintor J. (2012). Silencing of P2Y(2) receptors reduces intraocular pressure in New Zealand rabbits. *Br. J. Pharmacol.* 165 1163–1172. 10.1111/j.1476-5381.2011.01586.x 21740413 PMC3346251

[B18] Martin-Gil A. Pintor J. (2010). P2Y2 nucleotide receptors increase the presence of aquaporin-1 in rabbit non-pigmented ciliary epithelial cells. *Purinergic Signal.* 6 S155.

[B19] Mitchell C. H. Carre D. A. Mcglinn A. M. Stone R. A. Civan M. M. (1998). A release mechanism for stored ATP in ocular ciliary epithelial cells. *Proc. Natl. Acad. Sci. U.S.A.* 95 7174–7178. 10.1073/pnas.95.12.7174 9618558 PMC22777

[B20] Morrison J. C. Acott T. S. (2003). “Anatomy and physiology of aqueous humor outflow,” in *Glaucoma: Science and Practice*, eds Morrison J. C. Pollack I. P. (New York: Thieme), 34–41.

[B21] Nicholas R. A. Watt W. C. Lazarowski E. R. Li Q. Harden K. (1996). Uridine nucleotide selectivity of three phospholipase C-activating P2 receptors: identification of a UDP-selective, a UTP-selective, and an ATP- and UTP-specific receptor. *Mol. Pharmacol.* 50 224–229. 8700127

[B22] Patel K. Barnes A. Camacho J. Paterson C. Boughtflower R. Cousens D. (2001). Activity of diadenosine polyphosphates at P2Y receptors stably expressed in 1321N1 cells. *Eur. J. Pharmacol.* 430 203–210. 10.1016/S0014-2999(01)01401-7 11711032

[B23] Perez de Lara M. J. Guzman-Aranguez A. De La Villa P. Diaz-Hernandez J. I. Miras-Portugal M. T. Pintor J. (2015). Increased levels of extracellular ATP in glaucomatous retinas: Possible role of the vesicular nucleotide transporter during the development of the pathology. *Mol. Vis.* 21 1060–1070. 26392744 PMC4558477

[B24] Perez de Lara M. J. Santano C. Guzman-Aranguez A. Valiente-Soriano F. J. Aviles-Trigueros M. Vidal-Sanz M. (2014). Assessment of inner retina dysfunction and progressive ganglion cell loss in a mouse model of glaucoma. *Exp. Eye Res.* 122 40–49. 10.1016/j.exer.2014.02.022 24631335

[B25] Pintor J. Martin-Gil A. Fonseca B. (2011). Activation of TRPV4 induces the release of diadenosine tetraphosphate to the aqueous humour. *Invest. Ophthalmol. Vis. Sci.* 52 2052.

[B26] Pintor J. Peral A. Hoyle C. H. Redick C. Douglass J. Sims I. (2002). Effects of diadenosine polyphosphates on tear secretion in New Zealand white rabbits. *J. Pharmacol. Exp. Ther.* 300 291–297. 10.1124/jpet.300.1.291 11752128

[B27] Pintor J. Peral A. Pelaez T. Martin S. Hoyle C. H. (2003). Presence of diadenosine polyphosphates in the aqueous humor: their effect on intraocular pressure. *J. Pharmacol. Exp. Ther.* 304 342–348. 10.1124/jpet.102.041368 12490610

[B28] Pintor J. Sanchez-Nogueiro J. Irazu M. Mediero A. Pelaez T. Peral A. (2004). Immunolocalisation of P2Y receptors in the rat eye. *Purinergic Signal.* 1 83–90. 10.1007/s11302-004-5072-5 18404404 PMC2096566

[B29] Schachter J. B. Li Q. Boyer J. L. Nicholas R. A. Harden T. K. (1996). Second messenger cascade specificity and pharmacological selectivity of the human P2Y1-purinoceptor. *Br. J. Pharmacol.* 118 167–173. 10.1111/j.1476-5381.1996.tb15381.x 8733591 PMC1909474

[B30] Soto D. Pintor J. Peral A. Gual A. Gasull X. (2005). Effects of dinucleoside polyphosphates on trabecular meshwork cells and aqueous humor outflow facility. *J. Pharmacol. Exp. Ther.* 314 1042–1051. 10.1124/jpet.105.085274 15947035

[B31] Tomarev S. I. (2001). Eyeing a new route along an old pathway. *Nat. Med.* 7 294–295. 10.1038/85432 11231625

[B32] Waldo G. L. Harden T. K. (2004). Agonist binding and Gq-stimulating activities of the purified human P2Y1 receptor. *Mol. Pharmacol.* 65 426–436. 10.1124/mol.65.2.426 14742685

